# Is telerehabilitation an effective maintenance strategy for patients with chronic obstructive pulmonary diseases: a systematic review

**DOI:** 10.1186/s42269-023-00980-8

**Published:** 2023-02-01

**Authors:** Demelum Uche-Okoye, Michael Nnaemeka Ajemba, Bendall Amy, Ebube Chinwe Arene, Chinemerem Henry Ugo, Ngozi Perpetua Eze, Ikenna Kelechi Anyadike, Uju Maryanne Onuorah, Chijioke Michael Chiwenite

**Affiliations:** 1 NHS Dumfries and Galloway, Dumfries, DG1 4AP UK; 2 University of Nicosia Medical School, Egkomi, Cyprus; 3 Cardiff University, Cardiff, UK; 4 University of Nigeria, Nsukka, Nigeria; 5 Michael Okpara University of Agriculture, Umuahia, Nigeria

**Keywords:** Telerehabilitation, Patient, Chronic obstructive pulmonary disease, Exercise

## Abstract

**Background:**

Pulmonary rehabilitation (PR) has proven to improve the physical and psychosocial function in patients with chronic obstructive pulmonary disease (COPD). However, the gains achieved during pulmonary rehabilitation diminish over time without an effective maintenance strategy. With several factors affecting access to pulmonary rehabilitation, calls for innovative models were made, which saw the emergence of studies exploring telerehabilitation (TR) as an alternative to traditional pulmonary rehabilitation models. Although there are current reviews exploring the effectiveness of telerehabilitation as an alternative for conventional PR, no review has considered telerehabilitation effectiveness in the long term. Hence, this review aims at examining the effectiveness of telerehabilitation following to pulmonary rehabilitation in patients with chronic obstructive pulmonary disease.

**Main body:**

A systematic review of the literature using CINAHL, MEDLINE, SCOPUS, Web of science PEDRO, AMED and EMBASE databases was conducted to assess the effectiveness of telerehabilitation following PR in patients with COPD. Health-related quality of life (HRQoL) and exercise capacity was maintained within 6–12 months of a TR maintenance programme. However, there was no significant increase in HRQoL and exercise capacity between the intervention and control groups in 6–12 months.

**Conclusions:**

This review suggests that a TR maintenance strategy effectively maintains benefits gained and may improve HRQoL and exercise capacity within 6–12 months for patients with COPD. Nonetheless, it is impossible to extrapolate the findings to the general population due to the paucity of included studies. Further high quality randomised controlled trials examining TR in the long-term is required in the future.

**Supplementary Information:**

The online version contains supplementary material available at 10.1186/s42269-023-00980-8.

## Background

According to the National Institute for Health and Care Excellence [NICE] 2021, chronic obstructive pulmonary disease (COPD) is an umbrella term for chronic bronchitis and emphysema. Although closely related, they are not synonymous with each other. While chronic bronchitis is characterised by hypersecretion and prolonged cough (Kesimer et al. 2017), emphysema is characterised by structural changes in the extracellular matrix and air space of the terminal bronchiole (Amariei et al. 2019). A predominant feature of COPD is a poorly reversible airway limitation and dyspnoea known to cause a decrease in physical activity (PA), health-related quality of life (HRQoL) and an increase in acute exacerbation (Vestbo et al. 2013; Alqahtani et al. 2020).

Pulmonary rehabilitation (PR) is an evidence-based intervention proven effective in chronic respiratory conditions as it adopts a holistic approach in managing respective conditions (Camp and Cheung 2018) and involves a multi-disciplinary team that focuses on improving both the physical and psychological function of patients with COPD whilst promoting long-term adherence to health-enhancing behaviour, quality of life (QoL) and exercise tolerance improvement (Hill 2006; Spruit et al. 2013; Anastasaki et al. 2019). The various components of PR include but are not limited to exercise training, patient education, dietary changes and behavioural interventions (Camp and Cheung 2018). Additionally, PR promotes self-management strategies to enable individuals to self-manage their conditions effectively (Bolton et al. 2013). An individualised programme tailored to the patient needs, must be administered by a multi-disciplinary team to ensure a successful PR programme.

For an extended period, COPD treatments mostly focused on the pharmacological improvement of airway obstruction (Alqahtani et al. 2020). However, within the last 2 decades, the systemic impact of COPD in the overall health of individuals with the condition has precipitated the development of various non-pharmacological treatments such as pulmonary rehabilitation (PR) to augment the medical management of COPD (Corhay et al. 2014).

Several international guidelines recommend PR for symptomatic patients with COPD when pharmacological interventions alone do not decrease symptoms (Qaseem et al. 2011; Vestbo et al. 2013). Despite the documented benefits of PR, the long-term benefits of PR are short-lived without an effective maintenance strategy (Ries et al. 2003; Bolton et al. 2013). Several studies have explored maintenance strategies such as community PR (Moullec et al. 2008; van Wetering et al. 2010) and home-based PR (Wijkstra et al. 1995; Holland et al. 2013); the effectiveness of these strategies were not well-established in the literature. In the same vein, the American thoracic society and European respiratory society (ATS) acknowledged the urgent need for the research and development of novel models of pulmonary rehabilitation that will provide accessible, evidence-based PR programs for individuals with COPD (Rochester et al. 2015).

Several studies have investigated the delivery of PR while employing telemedicine-based interventions (Holland and Cox 2017; Zanaboni et al. 2017). Telerehabilitation, a subset of telemedicine, has been defined as the alternative way of delivering rehabilitation services using information and communication technologies (Laver et al. 2020). A systematic review by Chan et al. (2016) synthesised evidence on how telemonitoring and TR affected the quality of life (QOL) and exercise capacity of patients with COPD short term. The results show that TR provided similar results with usual care defined as traditional cardiac rehabilitation and PR. More so, the review was not specific to COPD and included patients with cardiovascular conditions. Finally, maintenance of benefits was neither mentioned nor investigated by the authors. A Cochrane review by Cox et al. (2020) explored the effects of TR in chronic respiratory diseases with results showing that TR achieves similar results as a traditional PR programme. The authors noted that 99% of the subject participants were patients with COPD. However, the findings obtained from this review should be treated with caution as a limited number of studies was included in the review and the small number of participants. None of those reviews mentioned above explored TR long-term or as a maintenance strategy following PR. Hence, even though the TR in COPD management is safe and feasible, it is still unclear if the benefits achieved after an outpatient’s PR programme can be maintained after 6–12 months with a TR maintenance programme.

Therefore, this review aims to explore the effectiveness of a < 6 months maintenance TR programme following a PR programme.

## Main text

Healthcare research informs the decisions and guidelines utilised in clinical practice. However, as the body of evidence grew, it became imperative to have a rigorous and systematic synthesis of available evidence (Aromataris and Pearson 2014). Historically, systematic reviews started showing up in health literature around the 1970s and 1980s with the advent of evidence-based healthcare and have continued to evolve till now (Bastian et al. 2010). Systematic reviews are rudimentary in evidence-based healthcare and provide the highest level in the hierarchy of evidence in research (Aromataris et al. 2015). Hence, a systematic review aims at providing a comprehensive, unbiased synthesis of evidence using transparent methods. It is methodological and utilises predetermined methods that appraise, summarise, and synthesise available primary research (Cajal et al. 2020). In order to maintain the same rigour and standard of primary research being reviewed, a pre-planned set of protocols to be peer-reviewed at the outset is published just like the research proposal (Jones and Evans 2000). According to JBI, this stage is perhaps the most important step as the planning and thoughtfulness put in this process ensures the review is well-defined, rigorous and reduces the risk of bias (Boland et al. 2017; Jordan et al. 2019).

### Review question

Is telerehabilitation an effective maintenance strategy following pulmonary rehabilitation for patients with chronic obstructive pulmonary diseases (COPD)? A systematic review.

## Methods

This systematic review followed a published protocol (CRD42022364398) and was conducted using the Preferred Reporting Items for Systematic Review and Meta—Analysis (PRISMA) method. A PRISMA checklist is presented in Additional file 1.

Our inclusion criteria were:

- Studies addressing adults aged > 40 with a diagnosis of COPD, randomised controlled trials (RCT), quasi-experimental studies and cohort studies.
- Studies with participants who completed > 6-month telerehabilitation maintenance programme following PR and any study that measures the following primary outcomes: exercise capacity and health-related quality of life (HRQoL).

We excluded studies on adults with other chronic respiratory conditions, studies where the participants did not receive PR or received only telemonitoring, all non-English language studies, animal studies, qualitative studies and grey literature.

### Information sources

The following databases were searched from inception to 18 October 2022; MEDLINE, EMBASE, PEDro, AMED, Web of Science, CINAHL, JBI, Scopus.

### Search strategy

The following keywords were used for the initial search: Telerehabilitation* OR tele-rehabilitation OR Telemedicine OR “remote rehabilitation” OR Telerehab* AND COPD OR “chronic obstructive disease*”. Boolean logic ‘OR’ commands expanded the search, while ‘AND’ commands limited the search. Truncations was used to expand words with varied endings.

A librarian was involved with the search development to improve the search quality. Citation (forward and backwards) chaining ensured maximum capturing of relevant articles. Grey literature were not included. Finally, article authors were contacted when more information is required.

The search strategy developed for each database is provided in Additional file 2.

### Study selection

The Preferred Reporting Items for Systematic Reviews and Meta-analyses’ (PRISMA) was utilised. The first stage involved merging selected studies using reference management software such as Endnote to eliminate duplicates. The titles and abstracts were then screened based on the inclusion/exclusion criteria to remove irrelevant articles. This process was carried out by four reviewers. Full-text retrieval of the remaining relevant studies followed suit, after which each study was read in full to ensure it fits the eligibility criteria.

### Data extraction

The JBI data extraction form for experimental studies was utilised for this review. It was piloted with two included studies then modified accordingly to extract only data relevant to the review. This enables standardisation of the data extraction tool across all included studies ensuring consistency and accuracy of data extracted. Four authors independently extracted the following data from included studies: (see Additional file 3 for the completed data extraction form).

- Participants: settings, population, gender, sample size and groups
- Baseline characteristics: age, sex, BMI, FEV1 (L) FEV1 (%) FVC (L) FVC (%) FEV1/FVC
- Intervention and comparison groups: IG & CG (*n* = 94) 8 weeks ± 4 days, IG (*n* = 46) 12 months, CG (*n* = 48) 12 months
- Clinical outcomes measures: Exercise capacity, Quality of life (QoL).

### Methodological quality assessment

Four independent reviewers used a validated critical appraisal tool to ensure that the highest quality of evidence is included in the review. The Physiotherapy Evidence-based database (PEDro) scale was utilised. Only studies that met all the essential criteria set out by the four reviewers proceeded to the next stage.

### Data synthesis

Data were synthesised narratively due to the heterogeneity of the outcome measures of interest.

## Result

**Fig. 1:**
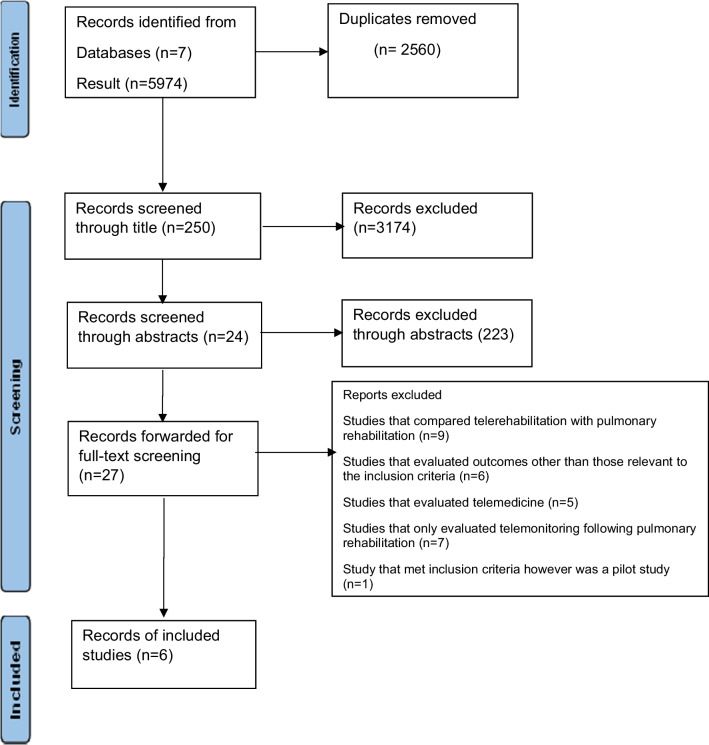
Prisma flow diagram

Five thousand nine hundred seventy-four articles were found during the search process, with 2640 duplicates removed. Following that, a title screening process of the remaining 3424 articles produced 250 articles, further filtered out by abstract screening. Back chaining of the reference lists did not reproduce any new studies meeting the inclusion criteria. Finally, 27 studies were read in full, and six articles were carried through to the critical appraisal stage. A modified PRISMA flow diagram illustrating the filtration process is shown in Fig. 1.

### Study characteristics

A summary of the interventions reported across the included studies is presented in Table 1. All the included studies were RCTs. Two studies were undertaken in Spain, while one was carried out in Greece, with the final study in Australia. This limits the generalisability of the studies as a greater proportion of studies was undertaken in Europe. Even one of the studies excluded during the critical appraisal stage was undertaken in Europe–Netherlands (Vorrink et al. 2016) with the study by Li et al. (2018) carried out in China. There was no variation in the population as all the participants were clinically stable which meant they did not have any acute exacerbations with COPD severity of II–IV based on the GOLD classification, correlating with recommendations by various international guidelines when offering patients with COPD PR (GOLD 2022; NICE 2022). The age range shows the lowest age range included was 18 years, with the highest being 85 years and a mean age of 51.5 years. Studies have shown that most patients present with COPD symptoms at an average age of 40 years (Izquierdo et al. 2021; Soriano et al. 2021). In addition, only about 2.8% of the COPD population are aged about 18–30 years (de Marco et al. 2007), with most of them presenting with α-1 antitrypsin deficiency (GOLD 2022).

**Table 1:** Characteristics of included studies

Study	Participants and demographics	Sample size	Intervention	Comparison	Outcome/measures
*Author/date* Galdiz et al. (2020) *Design and duration* 12-month RCT *Country* Spain	*Population* clinically stable patients diagnosed with moderate or severe COPD *Age* 18–75 years (average 63 years) *Gender* male and females *Groups* 2 (IG & CG)	*Total* 94 participants *IG* 46 *CG* 48	*PR programme* (8-weeks ± 4 days) Both groups (CG & IG) received a hospital out-patient PR programme 3x/7 with the session lasting approximately 2 h 30 min of arm weight lifting (1/2 kg and progressing) and 30 min bicycle ergometry, chest physiotherapy as indicated educational sessions targeted at the patients and family members or caregivers about self-management strategies, diet and diseases pathology) *TR programme (12-months*) Provision of a TR kit consisting of mobile phone, pulse oximeter, dumbells and bicycle ergometer. Communication is via a web-based platform with therapists monitoring vital signs and PA activity levels being input by patients Access to a system technician Individualised action plan with exercises similar to the PR programme and based on patient capacity	Usual care following clinical practice at the participating hospitals Patients were advised to at least 1 h per day	*Exercise capacity* 6MWT *QoL* CAT CRQ
*Author/date* Vasilopoulou et al. (2017) *Design and duration* 12-month RCT *Country* Greece	*Population clinically stable patients with moderate to severe COPD attending the out-patient clinic* *Age average age 67* *Gender male and female* *Groups 3*	*Total* 150 *IG 47* *CG* 50 *UC* 50	*PR Programme* (2 months) All groups (IG, CG & UC) received went through Supervised exercise training 3/7 days Cycling using the bicycle ergometer, resistance training for the upper and lower limbs, dietary advice, self-management techniques *IG-TR Programme* (12 months) Lenovo tablet, a wireless device measuring lung function (spirometry), pedometer, a video demonstrating exercises are installed on the tablet Tablet also has breathing techniques and educational leaflets on COPD, anxiety and depression Participants record vital signs on the web platform via the tab Upper and lower limb exercises, walking drills with exercises based on patient capacity The patients have access to a call centre (pulmonologist) in case of severe complications	CG—*Hospital-based maintenance rehabilitation programme* (12-months) Aerobic and resistance exercise 2/7 days similar to that of the PR programme. Consisted of 96 sessions performed *UC* Based on usual practice in Greece Optimal pharmacotherapy according to the GOLD guideline, vaccination, oxygen therapy	*Exercise capacity* 6MWT *QoL* SGRQ CAT mMRC
*Author/date *Jimenez-Reguera et al. (2020) *Design and duration 12—RCT* *Country Spain*	*Population* clinically stable COPD patients with GOLD classification II, III, IV *Age* 55–85 years *Gender* male and female *Groups* 2	*Total 44* *IG 20* *CG 24*	*Initial PR programme* (8-weeks) Based on the Spanish society of pulmonology and thoracic surgery, hospital protocols Muscle training, respiratory physiotherapy, education relevant to COPD *TR programme* (10-months) Mobile phone with web-based app (HappyAir) with two main parts educational part about COPD then data collection about PA and the condition App reminds participants to record medication intake, exercise duration, warning signs Patients were advised to perform physical activity and breathing exercises daily	*CG (10-months)* Patients are advised to engage in PA and breathing exercises	*Exercise capacity* 6MWT *QoL* CAT SQRQ Euro-5D
*Author/time* Wootton et al. (2018) *Design and duration* 12—RCT *Country* Sydney, Australia	*Population clinically stable COPD patients with GOLD classification II, III, IV* *Age 55–85 years* *Gender male and female* *Groups 2*	*Total 95 participants* *IG 49* *CG 46*	Subjects participated in a PR programme before randomisation *IG & CG Supervised exercise training (2 months)* Supervised walking training with usual medical care *IG-TR programme* (12 months) Motivation sessions and health coaching with the site coordinator prior to start of the intervention Telephone contacts with physiotherapist with structured questions and advice about overall health, diets, exercises and self-management strategies and PA goals for the next 2–4 weeks before the next session Encouraged to engage in PA at 3/7 Patients are asked to record their step counts and exercise duration in a diary	CG—12 months Unsupervised walking exercise 3/7 days	*Exercise capacity* 6MWT ISWT ESWT *QoL* SGRQ CRQ

All the included studies showed a significant difference in the percentage of men to women, with men making up a higher percentage of the population. All the participants were either current smokers intending to quit or ex-smokers, correlating with various studies that smoking is a major risk factor of COPD (Laniado-Laborín 2009; Halpin et al. 2019; Salvi et al. 2020). Only Galdiz et al. (2020) had participants who were non-smokers, but then evidence estimates 20% of the population with COPD has never smoked (Lamprecht et al. 2011). Although most of the participants were smokers, there appears to be a smoking cessation trend, with two studies showing a majority of the participants who have quit smoking (Vasilopoulou et al. 2017; Jimenez-Reguera 2020), reflecting on international recommendations about the influence of smoking cessation on COPD (British thoracic society [BTS] 2022; NICE 2020; GOLD 2022).

TR was delivered through two different forms by the included studies. A web-based platform installed on a mobile device was utilised by Galdiz et al. (2020), Vasilopoulou et al. (2017) and Jimenez-Reguera et al. (2020), while Wootton et al. (2018) utilised phone calls only to deliver TR. None of the interventions involved real-time remote telemonitoring except Vasilopoulou et al. (2017), where the participants were equipped with a multimodal device for spirometry and oxygen saturation measurement. All the other participants had to input data collected manually onto a web platform or in a diary, as in the case of Wootton et al. (2018).

Only Jimenez-Reguera et al. (2020) had the participants engaging in the TR maintenance programme at a higher frequency than the initial PR programme, similar to the frequency of several maintenance programmes (Ries et al. 2003; Bernocchi et al. 2018; Spencer and McKeough 2019). Galdiz et al. (2020) and Wootton et al. (2017) had the intervention group engaging at a frequency that is equivalent to the frequency of the initial PR programme, while Vasilopoulou et al. (2017) did not state the frequency as the participants engaged in the TR programme. A recent systematic review by Malaguti et al. (2021) found that the duration of a maintenance PR programme ranges from 4 weeks to 36 months. As shown in Table 1, all the included studies engaged in a TR maintenance programme for a 12-months duration except from Jimenez-Raguera et al. (2020), where participants underwent a 10-month TR maintenance programme. Physiotherapists oversaw the TR maintenance programme except in the study by Vasilopoulou et al. (2017), where the authors did not specify but noted healthcare professionals delivered the TR programme.

### Study Outcomes

The primary outcomes predetermined during the planning stage were exercise capacity and HRQoL. Table 1 shows all four included studies measured all the pre-set outcomes. However, there is a variation in the outcome measures for HRQoL as multiple outcome measures were used to measure one outcome. However, this is not unusual for conditions such as COPD, where an individual’s health status cannot be quantified adequately with a single outcome measure (Vestbo et al. 2013; Vickerstaff et al. 2021). HRQoL and QoL are often used interchangeably. However, QoL is a broad term that covers all aspects of human life. On the other hand, HRQoL is focused on the effect of the diseases and intervention on the QoL of the individual (Lin et al. 2013). The outcomes for HRQoL employed by the studies were SGRQ, CRQ, and CAT, proven valid and reliable (Reda et al. 2010; Ringbaek et al. 2012; Gupta et al. 2014). Furthermore, they represent the outcome measures used in COPD research to assess QOL (Ringbaek et al. 2012). Two of the studies used both SGRQ and CAT to assess HRQoL (Vasilopoulou et al. 2017; Jimenez-Reguera et al. 2020), Galdiz et al. (2020) used both CRQ and CAT, while Wootton et al. (2018) used SGRQ and CRQ. In addition, Galdiz et al. (2020) and Jimenez-Reguera et al. (2020) assessed QoL with the SP-36 and EuroQOL-5D tools, respectively.

Exercise capacity was assessed with the 6MWT only except Wootton et al. (2018) that employed two more outcome measures, ISWT and ESWT; therefore, little variability exists between the outcome measures for exercise capacity. Although the 6MWT may not be advantageous for individuals with better exercise tolerance, it is a valid and reliable outcome measure of functional capacity in cardiopulmonary patients (Ghofraniha et al. 2015).

### Reported biases

Some degree of limitation exists in the methodology of synthesis in terms of bias. For instance, the theoretical model was subjective as its best, the selection and grouping of the components of the study were equally subjective to an extent.

Due to the time constraint associated with this review, it was impossible to double the data extraction stage. The paucity of the included studies greatly affects the generalisability; therefore, the findings obtained in this review will not inform clinical practice. Rather, it will identify gaps and make recommendations for future research. Ongoing studies on the subject area show current research is ongoing on TR as an alternative to PR, especially with the pandemic and the need for social distancing. Although research into better technology to improve TR is currently underway, TR is still in its early stage of implementation in clinical practice worldwide (Alexander 2020).

## Discussion

There is currently an abundance of studies relating to TR as an alternative to PR in the short term; however, its long-term effectiveness as a maintenance strategy has not fully been explored.

A robust search strategy was formulated with the guidance of the subject librarian, as recommended by the Cochrane Handbook (Higgins et al. 2019). Following the formulation of the search strategy, a comprehensive search of key databases was carried out by the reviewer. The search process reproduced 3934 articles after deduplication. However, only six studies were included in the review. Initially, a substantial amount of evidence on TR in the long-term was found; however, on close inspection, it was discovered that some were duplicates while others did not meet the inclusion criteria as set out in the review protocol. Subsequently, following critical appraisal, four studies were taken to the data extraction and synthesis stage. Coincidentally, all the included studies were RCTs; however, the heterogeneity of the study outcome measures, and the paucity of the included studies precipitated narrative synthesis rather than meta-analysis for data synthesis.

This systematic review identified, included, and evaluated four studies on the effectiveness of a > 6 months TR maintenance programme as a maintenance strategy following PR. Although the studies show that intervention effectively preserved the benefits gained following PR, there is limited evidence of its superiority to other PR maintenance programmes (hospital-based maintenance and no care). In the same vein, the results of the included studies showed that there were no improvements across the outcomes assessed (HRQoL and exercise capacity) between groups over 6–12 months.

A systematic review by Imamura et al. (2020) found a medium-frequency maintenance programme, as seen in Wootton et al. (2018), where the participants were advised to engage in the intervention 2–3 times per week effective in maintaining exercise capacity but not HRQoL. The outcome measures utilised to assess exercise capacity were the 6MWT and the incremental shuttle walking test (ISWT), while the SGRQ assessed HRQOL. Although the variability displayed in the frequency of the included studies' maintenance programmes is similar to that seen in other studies (Beauchamp et al. 2013; Malaguti et al. 2021), it is difficult to determine the optimal frequency of maintenance programmes. Several studies have implicated individual perception of self, social/environmental factors, acute exacerbations, intrinsic motivation, structure/delivery of maintenance programmes as some of the determinants of long-term adherence (McAuley et al. 2007; O’Donoghue et al. 2018; Puggina et al. 2018). As witnessed in this current review, Wootton et al. (2018) had the IG receiving motivational sessions and health coaching, possibly explaining why the IG saw a lower drop-out rate while still maintaining benefits gained from baseline to the twelfth month. Maintenance programmes should not be a *one size fits all*. As seen with most of the included studies where the intervention was tailored to patient’s needs, it is important they are adapted to individual circumstances, considering environmental, socioeconomic, and individual health factors (Bogerd et al. 2011; McNamara et al. 2013). In contrast, this may pose a major challenge in determining the optimal frequency of a maintenance programme.

TR can be delivered asynchronously (delayed), synchronously (real-time) or with a combination of the two delivery modes (Hill and Breslin 2016). A scoping review by Morimoto et al. (2022) evaluating the theoretical approach and functional features of web platforms for TR in chronic diseases found web platforms to be more effective in delivering asynchronous correspondence than synchronous correspondence. A major reason for this is the ability of web platforms to display and unify multiple information available to all stakeholders, thereby decreasing the hurdle of participating in one's rehabilitation or a need for a different system as seen in real-time technology (Kloek et al. 2017; Cox and Holland 2019). However, this deviates from recent advancements in technology where telemonitoring systems are increasingly utilised to collect real-time health-related data (Lundell et al. 2015; Bourbeau and Farias 2018). As seen with several studies that utilised a web platform, it appears an asynchronous communication is better suited to improving adherence and promoting self-management than synchronous communication (Tabak et al. 2014; Zanaboni et al. 2016). Self-monitoring is known as one of the factors leading to improved maintenance (Busby et al. 2014). Hence, it may benefit studies to utilise self-monitoring devices such as wrist-worn activity trackers.

Similarly, recent studies have highlighted its role in optimising self-management, motivation and behavioural tracking amongst various age groups (Puri et al. 2017; Ellingson et al. 2019; Liao et al. 2020). In support, several qualitative studies have also shown patients’ participation in the daily registration of their symptoms and PA sessions provided them control over their health and increased their drive to engage in their sessions (Dinesen et al. 2011; Hoaas et al. 2016).

### Limitations

Several limitations were associated with this present review. Although the reviewers carried out a comprehensive search of various databases, the time constraint made it impossible to search grey literature, possibly leading to publication bias. The search restriction to only English language studies possibly introduced a language bias. In addition, to ensure reproducibility and reliability of findings, only a few studies were selected following the application of the inclusion and exclusion criteria, consequently reducing the number of studies reviewed and, as such, reducing the generalisability of findings.

Albeit the hierarchy of evidence of the included studies, RCT, none of the study participants was blinded to the intervention; similarly, only one study blinded the assessors, subsequently predisposing the review to selection bias, performance bias, and detection bias. Noteworthy, given the nature of the intervention, it was impossible to blind the participants and therapist as with many other physiotherapy interventions. Despite recommendations from Cochrane and JBI, the reviewer was unable to double the data extraction and data synthesis stage secondary to associated time constraints and feasibility, increasing the risk of substantial errors (Stephenson et al. 2020).

### Implication to clinical practice

The findings of this review suggest TR is effective in maintaining HRQoL and exercise capacity following PR for > 6 months. No adverse effects were reported for the use of intervention; however, there is no evidence that a > 6 months maintenance TR programme will improve HRQoL and exercise capacity. It appears the number of weeks the initial PR programme affected had a relationship with how long the benefit gained were maintained irrespective of a TR programme. For instance, it appears a 10-week PR appears to maintain benefits gained better than a 6-week PR programme in the long term (Steel et al. 2011; Cox et al. 2020).

It was observed that none of the studies included was carried out in the UK; the lack of research in the area of interest has affected the decisions such as policy changes, resource allocation and patient-related outcomes and so on. An audit statement released by the National Asthma and Chronic Obstructive Pulmonary Diseases Audit Programme [NACAP] shows that only about 1.3% of patients referred for PR are home-based- home visits or via TR (Singh et al. 2020). The majority of the intervention is delivered via home visits, with 40% by telephone calls and 2.3% by use of technology-based PR (videoconferencing) and 1.7% of other digital communication. Although the relevant NICE programmes mentioned maintenance, details about maintenance strategies were non-existent (NICE 2022).

In Greece, Vasilopoulou et al. (2017) reported very few PR services were available, with maintenance programmes completely non-existent. Furthermore, TR is more cost-effective than centre-based rehabilitation (Haesum et al. 2012; Frederix et al. 2020), with no adverse effect on delivering maintenance rehabilitative services via that route. The intervention also provides more options for clinicians when providing patient-centred care as it is imperative that care is tailored to individual patients' needs. Finally, the pandemic has opened an opportunity to explore future changes, not only making TR accessible but easily requested by patients. Hence, healthcare systems and stakeholders worldwide should consider re-evaluating and implementing this innovative service for long-term maintenance programmes.

### Future research

Recently, there has been a surge in the number of studies on the effectiveness of TR as an alternative to traditional models of PR short term; however, its long-term efficacy as a maintenance strategy is yet to be determined. Hence, there is a need for large and good quality multicentre RCTs on the long-term effectiveness of TR as increased effect size reduces associated bias. It is also important to explore the determinants and factors affecting a TR maintenance programme. This review highlights the need for more research into what constitutes an effective maintenance programme as it appears older studies on maintenance focused only on exercises for maintenance. Meanwhile, recent studies show exercises alone is not an effective maintenance strategy based on the nature of the condition.

There is a need for more homogenous studies on the TR maintenance programmes to standardise various patient groups based on the international classification of COPD (GOLD classification). It is possible that adapting evidence-based, individualised maintenance programmes for various treatment groups may be the way to go in the future. Following the COVID-19 pandemic, it appears people have become more familiarised with the use of online platforms coupled with the improvement of internet services. TR may become the first line of request for patients in COPD management. Finally, with recent advances in technology, further research on how to improve the TR experience and encourage self-management may improve the delivery of rehabilitative services.

## Conclusions

This review explored the effectiveness of a > 6 months TR as a maintenance strategy following PR. The possible impact on two outcomes (HRQoL and exercise capacity) was equally evaluated. The findings reveal a need for further research as the paucity of the included studies and the fact that the study was limited to mostly Europe and Australia limit the generalisability of the study findings.

## Supplementary Information

**Additional file 1.** PRISMA Checklist.

**Additional file 2.** Search strategy.

**Additional file 3.** Data extraction form.

## Data Availability

All data analysed during this study are included in this article and its additional file.

## Abbreviations

SGRQ: Saint George’s respiratory questionnaire
CRQ: Chronic respiratory questionnaire
BMI: Body mass index
FEV: Forced expiratory volume
FVC: Forced vital capacity
ISWT: Increment shuttle walking test
