# Supplementary material for: Is telerehabilitation an effective maintenance strategy for patients with chronic obstructive pulmonary diseases: a systematic review
Source: Bull Natl Res Cent. 2023 Feb 1;47(1):13. doi: 10.1186/s42269-023-00980-8 (PMC9890431; doi:10.1186/s42269-023-00980-8)
Supplement: Supplementary file 2 — Additional file 2. Search strategy. [file 42269_2023_980_MOESM2_ESM.docx]

**Additional file 2: Search strategy**

**CINAHL search**

| **Search** | **Keywords** | **Results** |
| --- | --- | --- |
| **S1** | “Chronic airway obstruction “ | 5566 |
| **S2** | “Chronic bronchitis” | 2104 |
| **S3** | “Chronic lung diseases” | 4588 |
| **S4** | “Chronic obstructive airway diseases” | 16800 |
| **S5** | “Chronic obstructive bronchitis” | 447 |
| **S6** | COPD OR “chronic obstructive pulmonary disease” | 26961 |
| **S7** | “Chronic obstructive bronchopulmonary diseases” | 12 |
| **S8** | COAD | 17276 |
| **S9** | “Pulmonary emphysema” | 3002 |
| **S10** | “Chronic obstructive respirat* “ | 702 |
| **S11** | “Chronic respiratory tract disease” | 4982 |
| **S12** | “Obstructive lung disease*” | 6477 |
| **S13** | “Obstructive respiratory disease*” | 657 |
| **S14** | S1 OR S2 OR S3 OR S4 OR S5 OR S6 OR S7 OR S8 OR S9 OR S10 OR S11 OR S12 OR S13 | 47463 |
| **S15** | Remote OR Remote consultation | 20923 |
| **S16** | Virtual OR “virtual reality” | 31905 |
| **S17** | “Remote rehabilitation” | 362 |
| **S18** | “Virtual rehabilitation” | 1119 |
| **S19** | Telerehab* | 774 |
| **S20** | “Web consult*” | 115 |
| **S21** | “Video consultation” | 580 |
| **S22** | Telemedicine | 34147 |
| **S23** | Telehealth | 11192 |
| **S24** | Tele-rehab* | 380 |
| **S24** | S15 OR S16 OR S17 OR S18 OR S19 OR S20 OR S21 OR S21 OR S22 OR S23 | 73725 |
| **S25** | S14 AND S24 | 547 |

**Table 3.3 Summary of reference**

| **Databases** | **References located** |
| --- | --- |
| CINAHL | 547 |
| MEDLINE | 1061 |
| EMBASE | 1947 |
| PEDro | 20 |
| Web Of Science | 1199 |
| AMED | 51 |
| SCOPUS | 1215 |
| Total | 5974 |
