# Supplementary material for: Is telerehabilitation an effective maintenance strategy for patients with chronic obstructive pulmonary diseases: a systematic review
Source: Bull Natl Res Cent. 2023 Feb 1;47(1):13. doi: 10.1186/s42269-023-00980-8 (PMC9890431; doi:10.1186/s42269-023-00980-8)
Supplement: Supplementary file 3 — Additional file 3. Data extraction form. [file 42269_2023_980_MOESM3_ESM.docx]

**Additional file 3: Data extraction form**

**Vasilopoulou et al. (2017)**

| **General information** | | | | | | | | | | | | | | | |
| --- | --- | --- | --- | --- | --- | --- | --- | --- | --- | --- | --- | --- | --- | --- | --- |
| **Reviewers** | | UD, MNA, UCH, EPN | | | | | | | | | | | | | |
| **Date extracted** | | 07/10/2022 | | | | | | | | | | | | | |
| **Author** | | Vasilopoulou et al. | | | | | | | | | | | | | |
| **Year** | | 2017 | | | | | | | | | | | | | |
| **Journal** | | European respiratory | | | | | | | | | | | | | |
| **Trial Number** | | NCT026187746 | | | | | | | | | | | | | |
| **Funding** | | General Secretariat for Research and Technology and the European Union *via* the National Strategic Reference Framework (NSRF 2007-2013). | | | | | | | | | | | | | |
| **Ethical approval** | | Sought form the scientific board of clinical studies at Sotrina Hospital when the study protocol was submitted | | | | | | | | | | | | | |
| **Study methods** | | | | | | | | | | | | | | | |
| **Research design** | | a multi-centre RCT | | | | | | | | | | | | | |
| **Statistical tests** | | One-way ANOVA, two-way ANOVA, Shapiro Wilk test | | | | | | | | | | | | | |
| **Participants** | | | | | | | | | | | | | | | |
| Settings | | Greece | | | | | | | | | | | | | |
| Population | | Clinically stable COPD patients with average aged >40 years | | | | | | | | | | | | | |
| Sample size | | 150 (power of 0.90 using an alpha significance level of 0.05 (two-sided)) | | | | | | | | | | | | | |
| Gender | | Male and females | | | | | | | | | | | | | |
| Groups | | IG- home based TR maintenance programme (n=50) CG- hospital-based maintenance programme (n=50) and UC-based on post-PR programme (n=50) | | | | | | | | | | | | | |
| **Baseline characteristic and demographics** | | | | | | | | | | | | | | | |
|  | | | | **IG (n=47)** | | | | **CG (n=50)** | | | | | **UC (50)** | | |
| **Sex (Male/Female)** | | | | 44/3 | | | | 38/12 | | | | | 37/13 | | |
| **Age (years)** | | | | 66.9 (9.6) | | | | 66.7 (7.3) | | | | | 64.0 (8.0) | | |
| **FEV_1_ (L)** | | | | 1.55 (0.80) | | | | 1.41 (0.48) | | | | | 1.42 (0.66) | | |
| **FEV_1_ %** | | | | 49.6 (21.9) | | | | 51.8 (17.3) | | | | | 51.7 (21.0) | | |
| **FVC (L)** | | | | 3.07(0.90) | | | | 2.70 (0.65) | | | | | 2.77 (0.81) | | |
| **FVC %** | | | | 80.7 (20.2) | | | | 78.4 (18.4) | | | | | 80.0 (20.3) | | |
| **FEV_1_/FVC %** | | | | 47.0 (14.1) | | | | 49.0 (12.7) | | | | | 51.9 (12.4) | | |
| **BMI (kg/m2)** | | | | 28.0 (5.3) | | | | 27.5 (5.0) | | | | | 26.4 (5.0) | | |
|  | | | |  | | | |  | | | | |  | | |
| **Intervention and comparison groups** | | | | | | | | | | | | | | | |
| **Intervention** | | | **Description** | | | | | | | | | | | | |
| 1. **IG & CG** (n=100) 8weeks   Initial PR | | | PR programme of supervised exercise 3/7 training sessions including 45mins on a bicycle ergometer, resistance training of the large muscle groups of the upper & lower limbs, dietary advice, breathing exercise and self-management exercise, | | | | | | | | | | | | |
| 1. **IG** (n=50) 12months | | | TR kit- Patients imputed their vital signs (HR & O2sat), daily steps, and lung function via wireless device fitted to Bluetooth technology and transmitting data to a Lenovo tablet. This data was reviewed 3-4/7 by different health care professionals. The exact exercise frequency largely depends on the patient's individual fitness level and data transmitted. Nil exercise equipment used | | | | | | | | | | | | |
| 1. **CG** (n=50) 12months | | | Aerobic and resistance training in the out-patient department 2/7(gym), dietary advice, breathing control and self-management techniques | | | | | | | | | | | | |
| 1. **UC** (n-50) 12months | | | Pharmacotherapy, oxygen therapy, vaccination, regular follow-up by a respiratory doctor | | | | | | | | | | | | |
| **Clinical outcomes measures** | | | | | | | | | | | | | | | |
| **Outcome description** | | | **Measures/scale** | | | | | | | | | | | | |
| Exercise capacity | | | Six-minute walk test (6MWT) | | | | | | | | | | | | |
| Quality of life (QoL) | | | St. George’s hospital respiratory questionnaire (SGRQ), CAT, mMRC | | | | | | | | | | | | |
| **Study results** (continuous data) | | | | | | | | | | | | | | | |
|  | **IG** | | | | | | **CG** | | | | | **UC** | | | |
| **Outcomes** | **Baseline** | | **2-month** | | **14-month** | **Baseline** | | | **2-months** | **14-month** | **Baseline** | | | **2-month** | **14-month** |
| **6MWT** | 389.1(91.**3)** | | 422.1(70.5)^¶^ | | 420.2(74.9)^¶^ | 385.1(80.3) | | | 423.0(70.5)^¶^ | 427.5(63.0)^¶^ | 384.8(90.2) | | | 382.4(80.3**)** | 339.9(110.1)^¶^ |
| **SGRQ** | 46.2(19.7) | | 42.2(19.2)^¶^ | | 38.4(20.5) ^¶^ | 43.5(16.7) | | | 35.5(15.7) ^¶^ | 33.6(16.5) ^¶^ | 44.1(16.6) | | | 44.7(16.9) | 50.2(17.7) ^¶^ |
| **CAT** | 17.6(8.1) | | 12.9(7.5) | | 13.0(7.3) | 15.7(5.6) | | | 13.2(5.8) ^¶^ | 11.8(5.6) ¶ | 15.8(4.9) | | | 16.1(6.2) | 20.9(6.7) ^¶^ |
| **mMRC** | 2.3(1.0) | | 1.8(0.9) ^¶^ | | 1.6(1.0) | 2.5(1.0) | | | 1.5(0.9) ^¶^ | 1.3(0.9) | 2.2(1.1) | | | 2.5(1.0) | 3.1(0.8) ^¶^ |
| **Authors conclusion** | | | | | | | | | | | | | | | |
| The home-based TR maintenance programme is an alternative to the hospital outpatient’s maintenance programme following PR. | | | | | | | | | | | | | | | |
| **Comments** | | | | | | | | | | | | | | | |
| QoL and exercise capacity were secondary outcomes in this study therefore analysis measuring between group differences was not carried out rather the focus was on acute exacerbation and adherence to the maintenance programmes. | | | | | | | | | | | | | | | |

**Jimenez-raguera et al. (2020)**

| **General information** | | | | | | | | | | | | |
| --- | --- | --- | --- | --- | --- | --- | --- | --- | --- | --- | --- | --- |
| **^Reviewer^** | | | UD, MNA, UCH, EPN | | | | | | | | | |
| **Date extracted** | | | ^08/10/2022^ | | | | | | | | | |
| **^Author^** | | | ^Jiménez-Reguera et al.^ | | | | | | | | | |
| **Year** | | | 2020 | | | | | | | | | |
| **Journal** | | | ^JMIR mHealth and uHealth^ | | | | | | | | | |
| **^Trial Number^** | | | ^NCT04479930^ | | | | | | | | | |
| **^Funding source^** | | | ^Board of Lovexair Foundation (HappyAir is an intellectual property of the Lovexair Foundation). EM one of the authors that supervised the data collection is one of the employees of Lovexair^ | | | | | | | | | |
| **^Ethics approval^** | | | ^approval gotten from the ethics committee of the hospitals involved in the study ( 12 de Octubre University Hospital, La Princesa University Hospital, and San Carlos Clinical University Hospital^ | | | | | | | | | |
| **Study methods** | | | | | | | | | | | | |
| **Research design** | | | A prospective longitudinal multi-centre RCT | | | | | | | | | |
| **Statistical analyses** | | | Kolmogorov-Smirnov normality test, Shapiro-wilk test, Wilcoxon and t-test, ANOVA | | | | | | | | | |
| **Participants** | | | | | | | | | | | | |
| **Setting** | | | Spain | | | | | | | | | |
| **Population** | | | Clinically stable patients diagnosed with a combination of COPD (II, II and IV GOLD class) aged of 55-85 years | | | | | | | | | |
| **Gender** | | | females and males only | | | | | | | | | |
| **Sample size** | | | 44 (power 80%) | | | | | | | | | |
| **Groups** | | | IG and CG | | | | | | | | | |
| **Baseline characteristics and demographics** | | | | | | | | | | | | |
|  | | | | | **IG** | | | | **CG** | | | |
| **Age (years)** | | | | | 68.1 (6.6) | | | | 68.1 (7.0) | | | |
| **Male %** | | | | | 9 (40.9) | | | | 13 (59.1) | | | |
| **Female %** | | | | | 8 (57.1) | | | | 6 (42.9) | | | |
| **BMI** | | | | | 26.50 (4.1) | | | | 26.07 (4.2) | | | |
| **FEV_1_ %** | | | | | 45.0 (15.3) | | | | 43.1 (13.6) | | | |
| **FVC %** | | | | | 78.6 (22.9) | | | | 72.6 (24.4) | | | |
| **FEV_1_/FVC (%)** | | | | | 49.06 (12.0) | | | | 44.5 (12.2) | | | |
| **Intervention and comparison groups** | | | | | | | | | | | | |
| **Intervention** | | | **Description** | | | | | | | | | |
| 1. IG & CG (n=44) 8weeks | | | Based on the Spanish society of pulmonology and thoracic surgery on exercise training, respiratory PT and education | | | | | | | | | |
| 1. IG (n=20) 10months | | | Web-based platform (HappyAir) with set weekly and monthly PA goals, patient monitoring of O_2_sats and step count, self-management trainings, educational content about exact pathology | | | | | | | | | |
| 1. CG (n=24) 10months | | | Community based programme where patients are advised to PA and breathing exercise daily | | | | | | | | | |
| **Clinical outcomes measures** | | | | | | | | | | | | |
| **Outcome description** | | | **Measures/scale** | | | | | | | | | |
| Exercise capacity | | | Six-minute walk test (6MWT) | | | | | | | | | |
| Quality of life (QoL) | | | COPD assessment test (CAT), SGRQ, EuroQOL-5D | | | | | | | | | |
| **Study results** (continuous data) | | | | | | | | | | | | |
| **IG** | | | | | | | | **CG** | | | | |
| **Outcomes** | | **2-months** | | **6-months** | | | **12-months** | **2-months** | | **6-months** | | **12-months** |
| 6MWT | | 378.4 (90.2) | | 348.2 (95.1) | | | 357.4 (112.5) | 362.6 (72.2) | | 326.4 (83.1) | | 339.9 (75.9) |
| SGRQ symptoms | | 43.1 (20.3) | | 34..7 (17.9) * | | | 39.5 (24.2) | 47.3 (19.4) | | 32.4 (17.3) * | | 37.5 (19) |
| SGRQ activities | | 60.3 (19.5) | | 60 (24) | | | 67.6 (22.4) | 65.7 (19.1) | | 66.6 (21.3) | | 71.4 (16.3) |
| SGRQ Impact | | 45.4 (9.4) | | 42.9(10.6) | | | 35.2(19.2) | 44.9 (16.6) | | 45.8 (16.8) | | 35.8 (16.1) * |
| SGRQ total | | 49.5 (11.1) | | 46.8 (13.3) * | | | 45.7 (19.8) | 50.4 (14.2) | | 49.9 (15.8) | | 46.8 (14) |
| EuroQOL-5D | | 0.5 (0.2) | | 0.6 (0.2) | | | 0.5 (0.2) | 0.5 (0.2) | | 0.5 (0.2) | | 0.5 (0.2) |
| CAT | | 14.3 (5.5) | | 12.1 (5.8) * | | | 14.1 (7.2) | 14.5 (7) | | 13.8 (7) | | 17.7 (7.4) |
| **Intragroup differences at 6-months and 12-months** | | | | | | | | | | | | |
| **6-months** | | | | | | | | **12-months** | | | | |
| **Outcomes** | **95% CI** | | | | | ***P*-value** | | **95% CI** | | | ***P*-value** | |
| **6MWT** | -34.5/88.3 | | | | | .38 | | -47.6/82.6 | | | .58 | |
| **SGRQ** | -10.2/13.9 | | | | | .77 | | -13.2/17.0 | | | .79 | |
| **CAT** | -5.8/3.1 | | | | | .53 | | -8.1/1.9 | | | .21 | |
| **Authors conclusion** | | | | | | | | | | | | |
| The development of a novel m-health app improved adherence, QOL and exercise capacity; however, the small sample size affects the generalisation of the results obtained | | | | | | | | | | | | |
| **Comments** | | | | | | | | | | | | |
| The authors did not report the intergroup and intragroup analysis of QOL and exercise capacity in a table or in the supplementary; however, they reported some significant between-group analysis in the text. This could be because these outcomes were secondary outcomes in the study. | | | | | | | | | | | | |

**Wootton et al. (2018)**

| **General information** | | | | | | | | | | | | |
| --- | --- | --- | --- | --- | --- | --- | --- | --- | --- | --- | --- | --- |
| **^Reviewer^** | | | UD, MNA, UCH, EPN | | | | | | | | | |
| **Date extracted** | | | ^8/10/2022^ | | | | | | | | | |
| **^Author^** | | | ^Jiménez-Reguera et al.^ | | | | | | | | | |
| **Year** | | | 2020 | | | | | | | | | |
| **Journal** | | | ^JMIR mHealth and uHealth^ | | | | | | | | | |
| **^Trial Number^** | | | ^NCT04479930^ | | | | | | | | | |
| **^Funding source^** | | | ^Board of Lovexair Foundation (HappyAir is an intellectual property of the Lovexair Foundation). EM one of the authors that supervised the data collection is one of the employees of Lovexair^ | | | | | | | | | |
| **^Ethics approval^** | | | ^approval gotten from the ethics committee of the hospitals involved in the study ( 12 de Octubre University Hospital, La Princesa University Hospital, and San Carlos Clinical University Hospital^ | | | | | | | | | |
| **Study methods** | | | | | | | | | | | | |
| **Research design** | | | A prospective longitudinal multi-centre RCT | | | | | | | | | |
| **Statistical analyses** | | | Kolmogorov-Smirnov normality test, Shapiro-wilk test, Wilcoxon and t-test, ANOVA | | | | | | | | | |
| **Participants** | | | | | | | | | | | | |
| **Setting** | | | Spain | | | | | | | | | |
| **Population** | | | Clinically stable patients diagnosed with a combination of COPD (II, II and IV GOLD class) aged of 55-85 years | | | | | | | | | |
| **Gender** | | | females and males only | | | | | | | | | |
| **Sample size** | | | 44 (power 80%) | | | | | | | | | |
| **Groups** | | | IG and CG | | | | | | | | | |
| **Baseline characteristics and demographics** | | | | | | | | | | | | |
|  | | | | | **IG** | | | | **CG** | | | |
| **Age (years)** | | | | | 68.1 (6.6) | | | | 68.1 (7.0) | | | |
| **Male %** | | | | | 9 (40.9) | | | | 13 (59.1) | | | |
| **Female %** | | | | | 8 (57.1) | | | | 6 (42.9) | | | |
| **BMI** | | | | | 26.50 (4.1) | | | | 26.07 (4.2) | | | |
| **FEV_1_ %** | | | | | 45.0 (15.3) | | | | 43.1 (13.6) | | | |
| **FVC %** | | | | | 78.6 (22.9) | | | | 72.6 (24.4) | | | |
| **FEV_1_/FVC (%)** | | | | | 49.06 (12.0) | | | | 44.5 (12.2) | | | |
| **Intervention and comparison groups** | | | | | | | | | | | | |
| **Intervention** | | | **Description** | | | | | | | | | |
| 1. IG & CG (n=44) 8weeks | | | Based on the Spanish society of pulmonology and thoracic surgery on exercise training, respiratory PT and education | | | | | | | | | |
| 1. IG (n=20) 10months | | | Web-based platform (HappyAir) with set weekly and monthly PA goals, patient monitoring of O_2_sats and step count, self-management training, educational content about exact pathology | | | | | | | | | |
| 1. CG (n=24) 10months | | | A community-based programme where patients are advised to PA and breathing exercises daily | | | | | | | | | |
| **Clinical outcomes measures** | | | | | | | | | | | | |
| **Outcome description** | | | **Measures/scale** | | | | | | | | | |
| Exercise capacity | | | Six-minute walk test (6MWT) | | | | | | | | | |
| Quality of life (QoL) | | | COPD assessment test (CAT), SGRQ, EuroQOL-5D | | | | | | | | | |
| **Study results** (continuous data) | | | | | | | | | | | | |
| **IG** | | | | | | | | **CG** | | | | |
| **Outcomes** | | **2-months** | | **6-months** | | | **12-months** | **2-months** | | **6-months** | | **12-months** |
| 6MWT | | 378.4 (90.2) | | 348.2 (95.1) | | | 357.4 (112.5) | 362.6 (72.2) | | 326.4 (83.1) | | 339.9 (75.9) |
| SGRQ symptoms | | 43.1 (20.3) | | 34..7 (17.9) * | | | 39.5 (24.2) | 47.3 (19.4) | | 32.4 (17.3) * | | 37.5 (19) |
| SGRQ activities | | 60.3 (19.5) | | 60 (24) | | | 67.6 (22.4) | 65.7 (19.1) | | 66.6 (21.3) | | 71.4 (16.3) |
| SGRQ Impact | | 45.4 (9.4) | | 42.9(10.6) | | | 35.2(19.2) | 44.9 (16.6) | | 45.8 (16.8) | | 35.8 (16.1) * |
| SGRQ total | | 49.5 (11.1) | | 46.8 (13.3) * | | | 45.7 (19.8) | 50.4 (14.2) | | 49.9 (15.8) | | 46.8 (14) |
| EuroQOL-5D | | 0.5 (0.2) | | 0.6 (0.2) | | | 0.5 (0.2) | 0.5 (0.2) | | 0.5 (0.2) | | 0.5 (0.2) |
| CAT | | 14.3 (5.5) | | 12.1 (5.8) * | | | 14.1 (7.2) | 14.5 (7) | | 13.8 (7) | | 17.7 (7.4) |
| **Intragroup differences at 6-months and 12-months** | | | | | | | | | | | | |
| **6-months** | | | | | | | | **12-months** | | | | |
| **Outcomes** | **95% CI** | | | | | ***P*-value** | | **95% CI** | | | ***P*-value** | |
| **6MWT** | -34.5/88.3 | | | | | .38 | | -47.6/82.6 | | | .58 | |
| **SGRQ** | -10.2/13.9 | | | | | .77 | | -13.2/17.0 | | | .79 | |
| **CAT** | -5.8/3.1 | | | | | .53 | | -8.1/1.9 | | | .21 | |
| **Authors conclusion** | | | | | | | | | | | | |
| The development of a novel m-health app improved adherence, QOL and exercise capacity; however, the small sample size affects the generalisation of the results obtained | | | | | | | | | | | | |
| **Comments** | | | | | | | | | | | | |
| The authors did not report the intergroup and intragroup analysis of QOL and exercise capacity in a table or the supplementary; however, they reported the significance between-group analysis in the text. This could be because these outcomes were secondary outcomes in the study. | | | | | | | | | | | | |
